# General Bahr-Esseen inequalities and their applications

**DOI:** 10.1186/s13660-017-1468-y

**Published:** 2017-08-18

**Authors:** István Fazekas, Sándor Pecsora

**Affiliations:** Faculty of Informatics, University of Debrecen, P.O. Box 400, Debrecen, 4002 Hungary

**Keywords:** 60F15, 60G50, Bahr-Esseen inequality, exponential inequality, Rosenthal inequality, strong law of large numbers, complete convergence, rate of convergence, acceptable random variables, weakly orthant-dependent sequences

## Abstract

We study the Bahr-Esseen inequality. We show that the Bahr-Esseen inequality holds with exponent *p* if it holds with exponent $q>p$ for the truncated and centered random variables. The Bahr-Esseen inequality is also true if the truncated random variables are acceptable. We then apply the results to obtain weak and strong laws of large numbers and complete convergence.

## Introduction

First, we recall the well-known Bahr-Esseen inequality. Let $1 \leq p \leq2$, and let $X_{n}$, $n=1,2,\dots$, be a sequence of independent random variables (r.v.s) with finite *p*th moment and mean zero (${\mathbb {E}}\vert X_{n}\vert^{p}<\infty$, ${\mathbb {E}}X_{n}=0$ for all $n=1,2,\dots$). Then

1.1 $$ {\mathbb {E}}\Biggl\vert \sum_{k=1}^{n} X_{k} \Biggr\vert ^{p}\leq c_{p,n} \sum _{k=1}^{n} {\mathbb {E}}\vert X_{k} \vert ^{p} $$

for all $n=1,2,\dots$, where $c_{p,n}\le2- n^{-1}$ (von Bahr and Esseen [1]). Inequality (1.1) is the *p*th von Bahr-Esseen moment inequality.

We remark that the *p*th von Bahr-Esseen moment inequality is obviously true for $0< p\le1$, that is, ${\mathbb {E}}\vert \sum_{k=1}^{n} X_{k} \vert ^{p}\leq\sum_{k=1}^{n} {\mathbb {E}}\vert X_{k}\vert^{p}$ for $0< p\le1$ and any sequence $X_{n}$, $n=1,2,\dots$, of random variables with finite *p*th moment.

Dharmadhikari and Jogdeo [2] proved the following inequality, which can be considered as an extension of the Bahr-Esseen inequality to the case $p>2$. Let $p \geq2$, and let $X_{n}$, $n=1,2,\dots$, be a sequence of independent random variables with finite *p*th moment and mean zero. Then (1.1) is satisfied with

$$ c_{p,n}= n^{p/2-1} \frac{p(p-1)}{2} \max\bigl\{ 1, 2^{p-3} \bigr\} \bigl[ 1+ 2p^{-1} D_{2m}^{(p-2)/2m} \bigr], $$

where the integer *m* satisfies $2m\le p < 2m+2$, and

$$ D_{2m} = \sum_{k=1}^{m} k^{2m-1} /(k-1)! \,. $$

In [3] the *p*th von Bahr-Esseen moment inequality was obtained for pairwise independent random variables and $1< p < 2$. The 2nd von Bahr-Esseen moment inequality is obvious for pairwise independent zero-mean random variables, and in [3] this fact is applied to prove the *p*th $(1< p<2)$ von Bahr-Esseen moment inequality. Analyzing the proof in [3], we can obtain the following result. Let $1 < p <2$, and let $X_{n}$, $n=1,2,\dots$, be a sequence of arbitrary random variables with finite *p*th moment and mean zero such that the 2nd von Bahr-Esseen moment inequality holds for the truncated and centered variables $X_{k} \mathbb{I}( \vert X_{k} \vert \le x )- {\mathbb {E}}X_{k} \mathbb{I}( \vert X_{k} \vert \le x )$, $k=1,2, \dots, n$, $x>0$, where $\mathbb{I}$ denotes the indicator function of a set. Then then the *p*th von Bahr-Esseen moment inequality is true for the random variables $X_{n}$, $n=1,2,\dots$, themselves.

Moreover, we can generalize the previous result using *q* instead of 2. That is, if $1 < p <q$ and the *q*th von Bahr-Esseen moment inequality holds for the truncated and centered variables, then the *p*th von Bahr-Esseen moment inequality holds true for the original random variables themselves.

However, there is another version of truncation. Given a r.v. *X* and a positive number *t*, we can use the following truncated r.v.:

1.2 $$ {}^{(-t)}{}X^{(t)} = -t \mathbb{I}\{X< -t \} + X \mathbb{I}\bigl\{ \vert X \vert \le t \bigr\} + t \mathbb{I}\{X> t \}. $$

The advantage of this truncation is that ${}^{(-t)}{}X^{(t)} = h(X) $ with an increasing real function *h*. We know that certain dependence conditions are inherited if the random variables are inserted into increasing functions. Therefore it is more important to know that the *q*th von Bahr-Esseen moment inequality for the truncated and centered variables ${}^{(-x)}{}X_{k}^{(x)}- {\mathbb {E}}{}^{(-x)}{}X_{k}^{(x)}$ implies the *p*th von Bahr-Esseen moment inequality for the original random variables $X_{k}$ themselves ($1 < p <q$). This fact is proved in our Theorem 2.1. We underline that in our Theorem 2.1 we do not assume any weak dependence condition for the random variables. We also emphasize that throughout the paper we use versions of truncation (1.2).

It is well known that certain exponential relations play a fundamental role in the proofs of asymptotic results for independent and weakly dependent random variables. A general form of such relations is included in the definition of acceptability. The r.v.’s $X_{1}, X_{2}, \dots, X_{n}$ are called acceptable if

1.3 $$ {\mathbb {E}}e^{\sum_{i=1}^{n} \lambda X_{i} } \le \prod_{i=1}^{n} {\mathbb {E}}e^{ \lambda X_{i}} $$

for any real number *λ* [4]. In Section 2.3, we show that a version of inequality (1.3) implies an exponential inequality; see Proposition 2.1. Then, using the exponential inequality, we obtain the Rosenthal inequality (Proposition 2.2). Finally, we will see that a version of inequality (1.3) implies the *p*th von Bahr-Esseen moment inequality; see Theorem 2.2. Applying Theorem 2.2, we obtain the von Bahr-Esseen’s moment inequality for WOD sequences (Theorem 2.3).

Important applications of moment inequalities are convergence theorems. In Section 2.4, we shall present laws of large numbers and complete convergence as consequences of our inequalities. According to the well-known Etemadi strong law of large numbers (SLLN), if $X_{1}, X_{2}, \dots$ are pairwise independent and identically distributed random variables with finite first moment, then

$$ \lim_{n\to\infty} \frac{X_{1} + \cdots+ X_{n}}{n} = {\mathbb {E}}X_{1} $$

almost surely [5]. Our Theorem 2.4 is an Etemadi style SLLN. In our theorem, instead of pairwise independence, we assume either (1.3) or (1.1) for the truncated r.v.s. Also, a well-known SLLN is the result of Csörgő, Tandori, and Totik [6]. There pairwise independent, but not identically distributed, r.v.s were considered. Our Theorem 2.5 is a new version of the Csörgő-Tandori-Totik SLLN. In our theorem, we replace pairwise independence with appropriate versions of (1.3) or (1.1). We also present a weak law of large numbers (WLLN); see Theorem 2.6.

The rate of convergence in laws of large numbers can be described by so-called complete convergence theorems. Classical complete convergence results are due to Hsu, Robbins, Erdős, Baum, and Katz; see [7]. First complete convergence results concerned probabilities; later, such results were proved for moments as well. The general form of complete moment convergence of the random variables $Y_{1}, Y_{2}, \dots$ is

$$ \sum_{n}^{\infty}a_{n} {\mathbb {E}}\bigl( \vert Y_{n} \vert / b_{n} - \varepsilon \bigr)_{+}^{q} < \infty $$

for all $\varepsilon>0$, where $(\cdot)_{+}$ denotes the positive part of a number. Here $Y_{n}$ is usually the partial sum of r.v.s. The classical paper dealing with complete moment convergence for independent r.v.s is [8]. Then several papers were devoted to the topic. In [9] it is shown that if certain moment inequalities are satisfied for the truncated r.v.s, then the complete moment convergence holds. In our paper, we prove the complete moment convergence if (1.3) is true for the truncated r.v.s (Theorem 2.7).

## Results and discussion

### Methods

In this paper, we apply truncations of random variables and then approximations of probabilities and moments. The combination of these methods enables us to obtain general versions of moment inequalities and convergence theorems.

### The von Bahr-Esseen moment inequality

In this subsection, we prove the following general theorem. If the von Bahr-Esseen moment inequality holds for *q* for the truncated and centered random variables, then it holds for the random variables themselves for any *p* with $1 < p < q$. We emphasize that there is no additional assumption on the dependence structure of the random variables. We mention that Theorem 2.1 in [3] is the Bahr-Esseens inequality for pairwise independent random variables. In our paper, we apply the method of the proof presented in [3]. However, as we use truncation (1.2) instead of $X_{k} \mathbb{I}( \vert X_{k} \vert \le x )$, our proof is shorter than that in [3].

#### Theorem 2.1

*Let* $1< p < q$. *Let* $X_{n},n=1,2,\dots$, *be a sequence of random variables with* ${\mathbb {E}}\vert X_{n}\vert^{p}<\infty$ *and* ${\mathbb {E}}X_{n}=0$ *for all* $n=1,2,\dots$. *Assume that*, *for any* $x>0$,

2.1 $$ {\mathbb {E}}\Biggl\vert \sum_{k=1}^{n} \bigl({}^{(-x)}{}X_{k}^{(x)}- {\mathbb {E}}{}^{(-x)}{}X_{k}^{(x)} \bigr) \Biggr\vert ^{q} \leq g_{q}(n) \sum _{k=1}^{n} {\mathbb {E}}\bigl\vert {}^{(-x)}{}X_{k}^{(x)}- {\mathbb {E}}{}^{(-x)}{}X_{k}^{(x)} \bigr\vert ^{q}. $$

*Then*

2.2 $$ {\mathbb {E}}\Biggl\vert \sum_{k=1}^{n} X_{k} \Biggr\vert ^{p}\leq f_{p,q}(n) \sum _{k=1}^{n} {\mathbb {E}}\vert X_{k} \vert ^{p}, $$

*where* $f_{p,q}(n)$ *depends only on* $g_{q}(n)$, *p*, *and* *q* (*a possible choice is* $f_{p,q}(n)= 5+ 2 c_{q} g_{q}(n) 2^{q} (\frac{q}{q-p} )^{2} $ *with* $c_{q}= 2^{q-1}$).

#### Proof

Let $V=\sum_{k=1}^{n}{\mathbb {E}}\vert X_{k}\vert^{p}$. If $V=0$, then $X_{k}=0$ a.s. for all $k=1,2,\dots, n$, so we may assume that $V\neq0$. For simplicity, $Z_{i}$ denotes the truncated random variable, that is, $Z_{i}= {}^{(-x^{1/p})}{}X_{i}^{(x^{1/p})}$, where *x* is an arbitrary positive number. For any $\varepsilon>1$,

2.3 $$ \begin{aligned}[b] {\mathbb {E}}\Biggl\vert \sum _{k=1}^{n}X_{k} \Biggr\vert ^{p} &= \int_{0}^{\infty} \mathbb{P}\Biggl\lbrace \Biggl\vert \sum _{k=1}^{n} X_{k} \Biggr\vert ^{p} > x \Biggr\rbrace \,\mathrm{d}x \\ &\leq(1+\varepsilon)V+ \int_{(1+\varepsilon)V}^{\infty} \mathbb{P}\Biggl\lbrace \Biggl\vert \sum _{k=1}^{n} X_{k} \Biggr\vert >x^{1/p} \Biggr\rbrace \,\mathrm{d}x \\ &\leq(1+\varepsilon)V+ \int_{(1+\varepsilon)V}^{\infty}\sum_{k=1}^{n} \mathbb{P}\bigl\lbrace \vert X_{k} \vert >x^{1/p} \bigr\rbrace \,\mathrm{d}x \\ &\quad+ \int_{(1+\varepsilon)V}^{\infty} \mathbb{P}\Biggl\lbrace \Biggl\vert \sum _{k=1}^{n} Z_{k} \Biggr\vert > x^{1/p} \Biggr\rbrace \,\mathrm{d}x \\ & =(1+\varepsilon)V+I_{1}+I_{2}. \end{aligned} $$

We see that

2.4 $$ I_{1}\leq\sum_{k=1}^{n} \int_{0}^{\infty} \mathbb{P}\bigl\lbrace \vert X_{k} \vert >x^{1/p} \bigr\rbrace \,\mathrm{d}x=\sum _{k=1}^{n}{\mathbb {E}}\vert X_{k} \vert ^{p}=V. $$

Using that ${\mathbb {E}}X_{k}=0$, we have that ${\mathbb {E}}X_{k} \mathbb{I}( \vert X_{k} \vert \leq x^{1/p} ) = -{\mathbb {E}}X_{k} \mathbb{I}( \vert X_{k} \vert > x^{1/p} )$, so we obtain

2.5 $$\begin{aligned} \sup_{x\geq(1+\varepsilon)V} x^{-1/p} \Biggl\vert \sum_{k=1}^{n} {\mathbb {E}}Z_{k} \Biggr\vert ={}& \sup_{x\geq(1+\varepsilon)V} x^{-1/p} \Biggl\vert \sum_{k=1}^{n}-{\mathbb {E}}X_{k} \mathbb{I}\bigl( \vert X_{k} \vert > x^{1/p} \bigr) \\ & {}+ x^{1/p}\mathbb{P}\bigl(X_{k} > x^{1/p} \bigr) - x^{1/p}\mathbb{P}\bigl(X_{k}< -x^{1/p} \bigr) \Biggr\vert \\ \leq {}&\sup_{x\geq(1+\varepsilon)V} x^{-1/p} \Biggl\vert \sum _{k=1}^{n}{\mathbb {E}}\vert X_{k} \vert \mathbb{I}\bigl( \vert X_{k} \vert > x^{1/p} \bigr)+ x^{1/p}\mathbb{P}\bigl( \vert X_{k} \vert > x^{1/p} \bigr) \Biggr\vert \\ \leq{}&2 \sup_{x\geq(1+\varepsilon)V} x^{-1/p} \sum _{k=1}^{n} {\mathbb {E}}\vert X_{k} \vert \mathbb{I}\bigl( \vert X_{k} \vert > x^{1/p} \bigr) \\ \leq{}&2 \sup_{x\geq(1+\varepsilon)V} x^{-1/p}\cdot x^{1/p-1}\sum _{k=1}^{n}{\mathbb {E}}\vert X_{k} \vert ^{p} \mathbb{I}\bigl( \vert X_{k} \vert > x^{1/p} \bigr) \\ \leq{}&2(1+\varepsilon)^{-1}V^{-1}\cdot V =2(1+ \varepsilon)^{-1}. \end{aligned}$$

Now we apply (2.5), and then, as $\varepsilon>1$, using Markov’s inequality, we obtain

2.6 $$ \begin{aligned}[b] I_{2}& = \int_{(1+\varepsilon)V}^{\infty} \mathbb{P}\Biggl\lbrace \Biggl\vert \sum _{k=1}^{n} Z_{k} \Biggr\vert > x^{1/p} \Biggr\rbrace \,\mathrm{d}x \\ &\leq \int_{(1+\varepsilon)V}^{\infty} \mathbb{P}\Biggl\lbrace \Biggl\vert \sum _{k=1}^{n} Z_{k} -\sum _{k=1}^{n} {\mathbb {E}}Z_{k} \Biggr\vert > x^{1/p} - \Biggl\vert \sum_{k=1}^{n} {\mathbb {E}}Z_{k} \Biggr\vert \Biggr\rbrace \,\mathrm{d}x \\ &\leq \int_{(1+\varepsilon)V}^{\infty} \mathbb{P}\Biggl\lbrace \Biggl\vert \sum _{k=1}^{n} [Z_{k}-{\mathbb {E}}Z_{k} ] \Biggr\vert > \bigl[1-2(1+\varepsilon)^{-1} \bigr]x^{1/p} \Biggr\rbrace \,\mathrm{d}x \\ &\leq \bigl[1-2(1+\varepsilon)^{-1} \bigr]^{-q} \int_{(1+\varepsilon )V}^{\infty}x^{-q/p}{\mathbb {E}}\Biggl\vert \sum _{k=1}^{n} [Z_{k}-{\mathbb {E}}Z_{k} ] \Biggr\vert ^{q} \,\mathrm{d}x \\ &\leq2 c_{q} g_{q}(n) \bigl[1-2(1+\varepsilon)^{-1} \bigr]^{-q}\sum_{k=1}^{n} \int_{(1+\varepsilon)V}^{\infty}x^{-q/p}{\mathbb {E}}\vert Z_{k} \vert ^{q} \,\mathrm{d}x \\ &= 2 c_{q} g_{q}(n) \bigl[1-2(1+\varepsilon)^{-1} \bigr]^{-q}\sum_{k=1}^{n} I_{2k}. \end{aligned} $$

In the last step we applied (2.1) and the $c_{q}$-inequality. Then, for a fixed *k*, $1\leq k\leq n$, we have

2.7 $$ \begin{aligned}[b] I_{2k} ={}& \int_{(1+\varepsilon)V}^{\infty}x^{-q/p}{\mathbb {E}}\vert Z_{k} \vert ^{q} \,\mathrm{d}x \\ ={}& \int_{(1+\varepsilon)V}^{\infty}x^{-q/p} \int_{0}^{x^{q/p}}\mathbb{P}\bigl\lbrace \vert X_{k} \vert ^{q}> y \bigr\rbrace \,\mathrm{d}y \,\mathrm{d}x \\ ={}& \int_{(1+\varepsilon)V}^{\infty}x^{-q/p} \int_{0}^{(1+\varepsilon )^{q/p}V^{q/p}}\mathbb{P}\bigl\lbrace \vert X_{k} \vert > y^{1/q} \bigr\rbrace \,\mathrm {d}y \,\mathrm{d}x\\ &{} + \int_{(1+\varepsilon)V}^{\infty}x^{-q/p} \int _{(1+\varepsilon)^{q/p}V^{q/p}}^{x^{q/p}}\mathbb{P}\bigl\lbrace \vert X_{k} \vert > y^{1/q} \bigr\rbrace \,\mathrm{d}y \,\mathrm{d}x \\ ={}&I_{21k}+I_{22k}. \end{aligned} $$

Again, using Markov’s inequality, we have

2.8 $$ \begin{aligned}[b] I_{21k} &=\frac{p}{q-p}(1+ \varepsilon)^{1-q/p}V^{1-q/p} \int _{0}^{(1+\varepsilon)^{q/p}V^{q/p}}\mathbb{P}\bigl\lbrace \vert X_{k} \vert > y^{1/q} \bigr\rbrace \,\mathrm{d}y \\ &\leq\frac{p}{q-p}(1+\varepsilon)^{1-q/p}V^{1-q/p} \int _{0}^{(1+\varepsilon)^{q/p}V^{q/p}}{\mathbb {E}}\vert X_{k} \vert ^{p}\cdot y^{-p/q}\,\mathrm{d}y \\ &=\frac{qp}{(q-p)^{2}}{\mathbb {E}}\vert X_{k} \vert ^{p}. \end{aligned} $$

For $I_{22k}$, we also get

2.9 $$\begin{aligned} I_{22k} &= \int_{(1+\varepsilon)^{q/p}V^{q/p}}^{\infty} \mathbb{P}\bigl\lbrace \vert X_{k} \vert > y^{1/q} \bigr\rbrace \int_{y^{p/q}}^{\infty}x^{-q/p}\,\mathrm{d}x \,\mathrm{d}y \\ &=\frac{p}{q-p} \int_{(1+\varepsilon)^{q/p}V^{q/p}}^{\infty}y^{p/q-1}\mathbb{P}\bigl\lbrace \vert X_{k} \vert > y^{1/q} \bigr\rbrace \,\mathrm{d}y \\ &\leq\frac{p}{q-p} \int_{0}^{\infty}y^{p/q-1}\mathbb{P}\bigl\lbrace \vert X_{k} \vert > y^{1/q} \bigr\rbrace \,\mathrm{d}y \\ &=\frac{q}{q-p}{\mathbb {E}}\vert X_{k} \vert ^{p}. \end{aligned}$$

Using relations (2.6)-(2.9), we get

2.10 $$ \begin{aligned}[b] I_{2} &\leq2 c_{q} g_{q}(n)\bigl[1-2(1+\varepsilon)^{-1}\bigr]^{-q} \biggl[\frac {qp}{(q-p)^{2}}+\frac{q}{q-p} \biggr]V \\ & = 2 c_{q} g_{q}(n)\bigl[1-2(1+\varepsilon)^{-1} \bigr]^{-q} \biggl(\frac {q}{q-p} \biggr)^{2} V. \end{aligned} $$

Summarizing (2.3), (2.4), and (2.10), we obtain

$$ {\mathbb {E}}\Biggl\vert \sum_{k=1}^{n}X_{k} \Biggr\vert ^{p} \leq \biggl\lbrace 2+\varepsilon+ 2c_{q} g_{q}(n) \bigl[1-2(1+\varepsilon )^{-1}\bigr]^{-q} \biggl(\frac{q}{q-p} \biggr)^{2} \biggr\rbrace V. $$

We can see that the function

$$ f(\varepsilon)= 2+\varepsilon+ 2c_{q} g_{q}(n) \bigl[1-2(1+ \varepsilon )^{-1}\bigr]^{-q} \biggl(\frac{q}{q-p} \biggr)^{2} $$

is positive and continuous on the interval $(1,\infty)$ and $\lim_{\varepsilon\rightarrow1^{+}}f(\varepsilon)=\lim_{\varepsilon\rightarrow\infty}f(\varepsilon)=\infty$. Therefore $f(\varepsilon)$ has a minimum on $(1, \infty)$. Let $f_{p,q}(n)=\inf_{1<\varepsilon<\infty}f(\varepsilon)$. We can see that $f_{p,q}(n)>3$, it depends only on $g_{q}(n)$, *p*, and *q*, and so (2.2) is proved. □

### Exponential inequalities and their consequences

In this subsection, we will see that if we assume that the exponential relation (1.3) is true for the truncated random variables, then we obtain an exponential inequality (Proposition 2.1), which implies Rosenthal’s inequality (Proposition 2.2) and von Bahr-Esseen’s moment inequality (Theorem 2.2).

Let $\eta_{1}, \eta_{2}, \dots, \eta_{n}$ be a sequence of r.v.s. Consider the condition

2.11 $$ {\mathbb {E}}e^{\sum_{i=1}^{n} \lambda\eta_{i} } \le g(n) \prod_{i=1}^{n} {\mathbb {E}}e^{ \lambda\eta_{i}}. $$

If condition (2.11) is satisfied for $g(n) =1$ and for all $\lambda \in \mathbb{R}$, then $\eta_{1}, \eta_{2}, \dots, \eta_{n}$ are called acceptable. It is easy to see that if (2.11) is true for $\eta_{1}, \eta_{2}, \dots, \eta_{n}$, then it is true for $\eta_{1}-a_{1}, \eta_{2}-a_{2}, \dots, \eta_{n}-a_{n}$ with any real numbers $a_{1}, \dots, a_{n}$; in particular, it is true for $\eta_{1}-{\mathbb {E}}\eta_{1}, \eta_{2}-{\mathbb {E}}\eta_{2}, \dots, \eta_{n}-{\mathbb {E}}\eta_{n}$.

Given a r.v. *X* and numbers $a< b$, we define the following (asymmetrically) truncated r.v.:

2.12 $$ {}^{(a)}{}X^{(b)} = a \mathbb{I}\{X< a \} + X \mathbb{I}\bigl\{ a\le X \le b \bigr\} + b \mathbb{I}\{X> b \}. $$

This truncation ${}^{(a)}{}X^{(b)}$ is an increasing function of *X*.

#### Proposition 2.1

*Let* $X_{1}, X_{2}, \dots, X_{n}$ *be a sequence of r*.*v*.*s*. *Assume that* (2.11) *is satisfied for any* $\lambda\in \mathbb{R}$ *and for* $\eta_{i}= {}^{(a_{i})}{}X_{i}^{(b_{i})}$ *with any* $a_{i}< b_{i}$, $i=1,2, \dots,n$. *Let* $d>0$ *be fixed*, *and let* $Y_{i}= {}^{(-d)}{}X_{i}^{(d)}- {\mathbb {E}}{}^{(-d)}{}X_{i}^{(d)}$, $i=1,2, \dots,n$, *be the truncated and centered r*.*v*.*s*. *Let* $S_{n} = \sum_{i=1}^{n} Y_{i}$, *and let* $B_{n} = \sum_{i=1}^{n} {\mathbb {E}}Y_{i}^{2}$ *be the sum of variances*. *Then*, *for any* $x>0$ *and* $t>0$, *we have*

2.13 $$ \mathbb{P}\bigl( \vert S_{n} \vert > x \bigr) \leq \mathbb{P}\Bigl(\max_{1\le i \le n} \vert Y_{i} \vert > t \Bigr) + 2 g(n) \exp \biggl(\frac{x}{t}- \frac{x}{t} \ln \biggl(1+ \frac{xt}{B_{n}} \biggr) \biggr). $$

#### Proof

We follow the classical ideas of [10] (see also [11] and [12]). For a real number $t>0$ and a r.v. *ξ*, let

$$\xi^{(t)} = \min\{ \xi, t\} $$

be the r.v. truncated from above. Let $\eta_{i}= Y_{i}^{(t)}$, $i=1,2, \dots, n$, denote our truncated r.v.s. Then $\eta_{i}$ are of the form ${}^{(a_{i})}{}X_{i}^{(b_{i})}-m_{i}$ for some $a_{i}< b_{i}$ and $m_{i}$, $i=1,2, \dots,n$. Therefore (2.11) is satisfied for $\eta_{i}= Y_{i}^{(t)}$. So usual argument (see [12]) gives

2.14 $$ \mathbb{P}\Biggl( \sum_{i=1}^{n} Y_{i}^{(t)} > x \Biggr) \leq g(n) \exp \biggl( \frac{x}{t}- \frac{x}{t} \ln \biggl(1+ \frac{xt}{B_{n}} \biggr) \biggr). $$

Inequality (2.11) is true for $\eta_{i}= (-Y_{i})^{(t)}$, $i=1,2, \dots,n $, so (2.14) is also true for the r.v.s $-Y_{1}, -Y_{2},\dots, -Y_{n}$. Applying (2.14) to both r.v.s $Y_{1}, Y_{2},\dots, Y_{n}$ and r.v.s $-Y_{1}, -Y_{2},\dots, -Y_{n} $, we get (2.13). □

Now we turn to Rosenthal’s inequality.

#### Proposition 2.2

*Let* $X_{1}, X_{2}, \dots, X_{n}$ *be a sequence of r*.*v*.*s*. *Assume that* (2.11) *is satisfied for any* $\lambda\in \mathbb{R}$ *and for* $\eta_{i}= {}^{(a_{i})}{}X_{i}^{(b_{i})}$ *with any* $a_{i}< b_{i}$, $i=1,2, \dots,n$. *Let* $d>0$ *be fixed*, *and let* $Y_{i}= {}^{(-d)}{}X_{i}^{(d)}- {\mathbb {E}}{}^{(-d)}{}X_{i}^{(d)}$, $i=1,2, \dots,n$, *be the truncated and centered r*.*v*.*s*. *Let* $S_{n} = \sum_{i=1}^{n} Y_{i}$, *and let* $B_{n} = \sum_{i=1}^{n} {\mathbb {E}}Y_{i}^{2}$ *be the sum of variances*. *Then*

2.15 $$ {\mathbb {E}}\vert S_{n} \vert ^{p} \le C_{1} {\mathbb {E}}\max_{1\le i \le n} \vert Y_{i} \vert ^{p} + 2 C_{2} g(n) B_{n}^{p/2}, $$

*where* $p>0$ *and* $C_{1}$, $C_{2}$ *depend only on* *p*.

#### Proof

It is known that the exponential inequality implies Rosenthal’s inequality; see, e.g., Theorem 3.1 in [12]. Therefore (2.13) implies (2.15). □

Now, we obtain the von Bahr-Esseen inequality.

#### Theorem 2.2

*Let* $1< p \le2$. *Let* $X_{n},n=1,2,\dots$, *be a sequence of random variables with* ${\mathbb {E}}\vert X_{n}\vert^{p}<\infty$ *and* ${\mathbb {E}}X_{n}=0$ *for all* $n=1,2,\dots$. *Assume that* (2.11) *is satisfied for any* $\lambda\in \mathbb{R}$ *and for* $\eta_{i}= {}^{(a_{i})}{}X_{i}^{(b_{i})}$ *with any* $a_{i}< b_{i}$, $i=1,2, \dots,n$. *Then*

2.16 $$ {\mathbb {E}}\Biggl\vert \sum_{k=1}^{n} X_{k} \Biggr\vert ^{p}\leq f_{p}(n) \sum _{k=1}^{n} {\mathbb {E}}\vert X_{k} \vert ^{p}, $$

*where* $f_{p}(n)$ *depends only on* $g(n)$ *and* *p*.

#### Proof

Let $d>0$ be fixed, and let $Y_{i}= {}^{(-d)}{}X_{i}^{(d)}- {\mathbb {E}}{}^{(-d)}{}X_{i}^{(d)}$, $i=1,2, \dots,n$, be the truncated and centered r.v.s. Let $S_{n} = \sum_{i=1}^{n} Y_{i}$ be their sum, and $B_{n} = \sum_{i=1}^{n} {\mathbb {E}}Y_{i}^{2}$ be the sum of variances. Then, by Proposition 2.2 with exponent 2 we have

2.17 $$ {\mathbb {E}}\Biggl( \sum_{i=1}^{n} Y_{i} \Biggr)^{2} ={\mathbb {E}}\vert S_{n} \vert ^{2} \le C g(n) B_{n} = \sum_{i=1}^{n} C g(n) {\mathbb {E}}Y_{i}^{2}. $$

So we obtained that the von Bahr-Esseen moment inequality holds for exponent 2 for the truncated and centered random variables. Therefore, by Theorem 2.1 it holds for the random variables themselves for any exponent *p* with $1 < p < 2$. So (2.16) is proved for $1 < p < 2$. For $p=2$, we use $d\uparrow\infty$ in (2.17). Then the dominated convergence theorem implies (2.16) if $p=2$. □

Now, we apply our results to widely orthant-dependent sequences. A sequence of r.v.s $X_{1}, X_{2}, \dots$ is said to be widely orthant-dependent (WOD) if, for any positive integer *n*, there exists a finite $g(n)$ such that, for any real numbers $x_{1}, \dots, x_{n}$, we have

2.18 $$ \mathbb{P}( X_{1} >x_{1}, X_{2} >x_{2}, \dots, X_{n}> x_{n}) \le g(n) \prod _{i=1}^{n} \mathbb{P}(X_{i} >x_{i}) $$

and

2.19 $$ \mathbb{P}( X_{1} \le x_{1}, X_{2} \le x_{2}, \dots, X_{n} \le x_{n}) \le g(n) \prod _{i=1}^{n} \mathbb{P}(X_{i} \le x_{i}); $$

see [13]. It is known that extended negatively orthant-dependent sequences, negatively orthant-dependent sequences, negatively superadditive dependent sequences, negatively associated and independent sequences are WOD; see [14]. We list a few known facts on WOD sequences.

If $X_{1}, X_{2}, \dots$ is a WOD sequence and the real functions $f_{1}, f_{2}, \dots$ are either all nondecreasing or all nonincreasing, then the sequence $f_{1}(X_{1}), f_{2}(X_{2}), \dots$ is also WOD. In particular, the truncated sequence ${}^{(a_{i})}{}X_{i}^{(b_{i})}$, $i=1,2, \dots$, is WOD. Moreover,

2.20 $$ {\mathbb {E}}e^{\sum_{i=1}^{n} \lambda X_{i} } \le g(n) \prod_{i=1}^{n} {\mathbb {E}}e^{ \lambda X_{i}} $$

for any real number *λ* and with $g(n)$ in (2.18)-(2.19). Now, we obtain the von Bahr-Esseen inequality for WOD sequences. We remark that the following theorem was obtained using a different setup in [14] (see Corollary 2.3 of [14]).

#### Theorem 2.3

*Let* $1< p \le2$. *Let* $X_{n},n=1,2,\dots$, *be a WOD sequence of random variables satisfying* (2.18) *and* (2.19). *Assume that* ${\mathbb {E}}\vert X_{n}\vert^{p}<\infty$ *and* ${\mathbb {E}}X_{n}=0$ *for all* $n=1,2,\dots$. *Then*

2.21 $$ {\mathbb {E}}\Biggl\vert \sum_{k=1}^{n} X_{k} \Biggr\vert ^{p}\leq f_{p}(n) \sum _{k=1}^{n} {\mathbb {E}}\vert X_{k} \vert ^{p}, $$

*where* $f_{p}(n)$ *depends only on* *p* *and* $g(n)$ *from inequalities* (2.18)-(2.19).

#### Proof

Because of the above-mentioned properties of WOD sequences, we can apply Theorem 2.2. □

### Convergence theorems

In this subsection, we prove general convergence theorems. We show that when the acceptability relation (2.11) is satisfied for the truncated random variables, then weak and strong laws of large numbers (WLLN, SLLN) and complete convergence hold without any further weak dependence assumption. As the proofs go through the Bahr-Esseen inequality, we can see that the validity of (2.16) for the truncated and centered random variables implies the above-mentioned asymptotic results.

We start with an Etemadi style SLLN.

#### Theorem 2.4

*Let* $X_{n},n=1,2,\dots$, *be a sequence of identically distributed r*.*v*.*’ satisfying* ${\mathbb {E}}X_{1}^{2}<\infty$ *and* ${\mathbb {E}}X_{1}=0$.

1. *Assume that* (2.11) *is satisfied with* $g(n)=C$ *for any* $\lambda\in \mathbb{R}$ *and for* $\eta_{i}= {}^{(a_{i})}{}X_{i}^{(b_{i})}$ *with any* $a_{i}< b_{i}$, $i=1,2, \dots$. *Then*
  2.22 $$ \lim_{n\to\infty} \frac{1}{n} \sum _{k=1}^{n} X_{k} =0 $$
  *with probability* 1.
2. *If*, *instead of* (2.11), *the Bahr*-*Esseen inequality is satisfied for the truncated and centered r*.*v*.*’*, *that is*, *if*
  2.23 $$ {\mathbb {E}}\Biggl( \sum_{i=1}^{n} \bigl( {}^{(a_{i})}{}X_{i}^{(b_{i})} - {\mathbb {E}}{}^{(a_{i})}{}X_{i}^{(b_{i})} \bigr) \Biggr)^{2} \leq C \sum_{i=1}^{n} {\mathbb {E}}\bigl({}^{(a_{i})}{}X_{i}^{(b_{i})} - {\mathbb {E}}{}^{(a_{i})}{}X_{i}^{(b_{i})} \bigr)^{2}, $$
  *then* (2.22) *is satisfied*.

#### Proof

First, we remark that, by Theorem 2.2, inequality (2.23) is always satisfied under the conditions of our theorem. We know that the original Etemadi SLLN is satisfied for pairwise independent r.v.s. However, analyzing the proof (see [5] or [15]), the only step where pairwise independence is applied is the use of inequality (2.23) with $a_{i}=0$, $b_{i}=i$ and with $a_{i}=-i$, $b_{i}=0$. □

A well-known SLLN for pairwise independent r.v.s is the result of Csörgő, Tandori, and Totik [6]. We show that Theorem 1 in [6] is valid if pairwise independence is replaced by an acceptability condition. We mention that in our theorem *p* is arbitrary with $1< p<2$, whereas $p=2$ in [6].

#### Theorem 2.5

*Let* $1< p<2$. *Let* $X_{n},n=1,2,\dots$, *be a sequence of r*.*v*.*’*. *Assume that*

2.24 $$ \sum_{m=1}^{\infty} {\mathbb {E}}\frac{ \vert X_{m} - {\mathbb {E}}X_{m} \vert ^{p}}{m^{p}} < \infty $$

*and*

2.25 $$ \frac{1}{n}\sum_{m=1}^{n} {\mathbb {E}}\vert X_{m} - {\mathbb {E}}X_{m} \vert \quad \textit{is bounded}. $$

1. *Assume that* (2.11) *is satisfied with* $g(n)=C$ *for any* $\lambda\in \mathbb{R}$ *and for* $\eta_{i}= {}^{(a_{i})}{}X_{i}^{(b_{i})}$ *with any* $a_{i}< b_{i}$, $i=1,2, \dots$. *Then*
  2.26 $$ \lim_{n\to\infty} \frac{1}{n} \sum _{m=1}^{n} (X_{m} - {\mathbb {E}}X_{m} ) =0 $$
  *with probability* 1.
2. *If*, *instead of* (2.11), *the Bahr*-*Esseen inequality is satisfied for the truncated and centered r*.*v*.*s*, *that is*, *if*
  2.27 $$ {\mathbb {E}}\Biggl\vert \sum_{i=1}^{n} \bigl({}^{(a_{i})}{}X_{i}^{(b_{i})} - {\mathbb {E}}{}^{(a_{i})}{}X_{i}^{(b_{i})} \bigr) \Biggr\vert ^{p} \leq C \sum_{i=1}^{n} {\mathbb {E}}\bigl\vert {}^{(a_{i})}{}X_{i}^{(b_{i})}- {\mathbb {E}}{}^{(a_{i})}{}X_{i}^{(b_{i})} \bigr\vert ^{p}, $$
  *then* (2.26) *is satisfied*.

#### Proof

By Theorem 2.2 inequality (2.27) is always satisfied under the conditions of our theorem. In the original proof (see [6]) the only step where pairwise independence is applied is the use of inequality (2.27) with with $a_{i}=0$, $b_{i}=\infty$ and with $a_{i}=-\infty$, $b_{i}=0$. □

It is known that in the case of nonidentically distributed random variables certain regularity conditions should be imposed for the moments or for the distributions (e.g., conditions (2.24) and (2.25)). Such a condition is the weak mean domination.

A sequence of r.v.s $Y_{n}$, $i=1,2,\dots$, is called weakly mean dominated (wmd) by the a r.v. *Y* if

2.28 $$ \frac{1}{n} \sum_{i=1}^{n} \mathbb{P}\bigl( \vert Y_{i} \vert > t \bigr) \le C \mathbb{P}\bigl( \vert Y \vert > t \bigr) $$

for all $t\ge0$ and $n=1,2,\dots$ (see Gut [16]).

We will often use the following lemma (see [17]).

#### Lemma 2.1

*Let the sequence* $Y_{n}$, $i=1,2,\dots$, *be weakly mean dominated by a r*.*v*. *Y*. *Let* $t>0$ *be fixed*. *Let* $f: [0,\infty) \to[0, \infty)$ *be a strictly increasing unbounded function with* $f(0) = 0$. *Then*

- 2.29 $$ \frac{1}{n} \sum_{i=1}^{n} {\mathbb {E}}\vert Y_{i} \vert \le C {\mathbb {E}}\vert Y \vert ; $$
- *the sequence* $f(|Y_{n}|)$, $i=1,2,\dots$, *is weakly mean dominated by the r*.*v*. $f(|Y|)$;
- *the truncated sequence* ${}^{(-t)}{}Y_{n}^{(t)}$, $i=1,2,\dots$, *is weakly mean dominated by the truncated r*.*v*. ${}^{(-t)}{}Y^{(t)}$;
- 2.30 $$ \frac{1}{n} \sum_{i=1}^{n} {\mathbb {E}}\vert Y_{i} \vert \mathbb{I}\bigl\{ \vert Y_{i} \vert > t \bigr\} \le C {\mathbb {E}}\vert Y \vert \mathbb{I}\bigl\{ \vert Y \vert > t \bigr\} . $$

The following theorem contains a WLLN and $L_{p}$-convergence.

#### Theorem 2.6

*Let* $1< p < 2$. *Let the sequence* $X_{n},n=1,2,\dots$, *be weakly mean dominated by a r*.*v*. *X* *with* ${\mathbb {E}}\vert X\vert^{p}<\infty$. *Assume that* ${\mathbb {E}}X_{n}=0$ *for all* $n=1,2,\dots$. *Assume that* (2.11) *is satisfied with* $g(n)=C$ *for any* $\lambda\in \mathbb{R}$ *and for* $\eta_{i}= {}^{(a_{i})}{}X_{i}^{(b_{i})}$ *with any* $a_{i}< b_{i}$, $i=1,2, \dots$. *Then*

2.31 $$ \lim_{n\to\infty} {\mathbb {E}}\Biggl\vert \frac{1}{n^{1/p}} \sum_{k=1}^{n} X_{k} \Biggr\vert ^{p} =0. $$

*Moreover*,

2.32 $$ \lim_{n\to\infty} \frac{1}{n^{1/p}} \sum _{k=1}^{n} X_{k} =0 $$

*in probability*.

#### Proof

Let $t>0$. Define

$${}^{(-\infty)}{}Z^{(-t)} =\min\{ -t, Z\}, \qquad {}^{(t)}{}Z^{(\infty )} = \max\{ t, Z\}. $$

As ${\mathbb {E}}X_{k} =0$, we have

2.33 $$ \begin{aligned}[b] {\mathbb {E}}\Biggl\vert \frac{1}{n^{1/p}} \sum_{k=1}^{n} X_{k} \Biggr\vert ^{p} \le{}& c {\mathbb {E}}\Biggl\vert \frac{1}{n^{1/p}} \sum _{k=1}^{n} \bigl( {}^{(-\infty )}{}X_{k}^{(-t)} - {\mathbb {E}}{}^{(-\infty)}{}X_{k}^{(-t)} \bigr) \Biggr\vert ^{p} \\ & {}+ c {\mathbb {E}}\Biggl\vert \frac{1}{n^{1/p}} \sum _{k=1}^{n} \bigl( {}^{(-t)}{}X_{k}^{(t)} - {\mathbb {E}}{}^{(-t)}{}X_{k}^{(t)} \bigr) \Biggr\vert ^{p} \\ & {}+ c {\mathbb {E}}\Biggl\vert \frac{1}{n^{1/p}} \sum _{k=1}^{n} \bigl( {}^{(t)}{}X_{k}^{(\infty)} - {\mathbb {E}}{}^{(t)}{}X_{k}^{(\infty)} \} \bigr) \Biggr\vert ^{p} \\ ={}& T_{1} + T_{2} + T_{3}, \end{aligned} $$

say. Applying Theorem 2.2, we obtain

$$ \begin{aligned}[b] T_{3} \le{}&\frac{c}{n} \sum _{k=1}^{n} {\mathbb {E}}\bigl\vert {}^{(t)}{}X_{k}^{(\infty )} - {\mathbb {E}}{}^{(t)}{}X_{k}^{(\infty)} \bigr\vert ^{p} \\ \le{}&\frac{c}{n} \sum_{k=1}^{n} {\mathbb {E}}\bigl\vert t \mathbb{I}\{X_{k} > t \} - {\mathbb {E}}t \mathbb{I}\{X_{k} > t \} \bigr\vert ^{p} \\ & {}+ \frac{c}{n} \sum_{k=1}^{n} {\mathbb {E}}\bigl\vert X_{k} \mathbb{I}\{X_{k} > t \} - {\mathbb {E}}X_{k} \mathbb{I}\{X_{k} > t \} \bigr\vert ^{p} \\ \le{}&\frac{c}{n} \sum_{k=1}^{n} t^{p} \mathbb{P}\{X_{k} > t \} + \frac{c}{n} \sum _{k=1}^{n} {\mathbb {E}}X_{k}^{p} \mathbb{I}\{X_{k} > t \} \\ \le{}&\frac{c}{n} \sum_{k=1}^{n} {\mathbb {E}}X_{k}^{p} \mathbb{I}\{X_{k} > t \} . \end{aligned} $$

Similarly,

$$ T_{1} \le\frac{c}{n} \sum_{k=1}^{n} {\mathbb {E}}\vert X_{k} \vert ^{p} \mathbb{I}\{X_{k} < -t \} . $$

Therefore, by (2.30),

$$ T_{1} + T_{3} \le\frac{c}{n} \sum _{k=1}^{n} {\mathbb {E}}\vert X_{k} \vert ^{p} \mathbb{I}\bigl\{ \vert X_{k} \vert > t \bigr\} \le c {\mathbb {E}}\vert X \vert ^{p} \mathbb{I}\bigl\{ \vert X \vert > t \bigr\} \le \frac{\varepsilon}{2} $$

for any fixed $\varepsilon>0$ if *t* is large enough, that is, $t\ge t_{\varepsilon}$, say. Now, applying Theorem 2.2 with exponent 2, we obtain

2.34 $$ \begin{aligned}[b] T_{2} &\le \frac{c}{n} \Biggl( \sum _{k=1}^{n} {\mathbb {E}}\bigl( {}^{(-t)}{}X_{k}^{(t)} - {\mathbb {E}}{}^{(-t)}{}X_{k}^{(t)} \bigr)^{2} \Biggr)^{p/2} \\ & \le\frac{c}{n} \bigl(n (2t)^{2} \bigr)^{p/2} = c t^{p} n^{p/2} /n . \end{aligned} $$

Let $t=t_{\varepsilon}$ and choose *n* large enough so that $c t_{\varepsilon}^{p} n^{p/2} /n \le\frac{\varepsilon}{2}$. Then $T_{2} \le\varepsilon/2$. □

#### Remark 2.1

Our Theorem 2.6 is similar to Theorem 3.1 of [3], where pairwise independent r.v.s were considered. We can see that in our theorem the weak mean domination assumption can be replaced by the *p*th uniform integrability assumption used in Theorem 3.1 of [3].

In the following theorem, we will see that if the acceptability condition (2.11) with $g(n) =C$ holds for the truncated random variables, then complete (moment) convergence results can be obtained. In particular, if the Bahr-Esseen inequality holds for the truncated and centered random variables, then complete (moment) convergence holds.

#### Theorem 2.7

*Let* $0< p < 2$, $1\le r < 2$, *and* $0 < \alpha<2$. *Let the sequence* $X_{n}$, $n=1,2,\dots$, *be weakly mean dominated by a r*.*v*. *X*. *Assume that* ${\mathbb {E}}X_{n}=0$ *for all* $n=1,2,\dots$. *Assume that* (2.11) *is satisfied with* $g(n)=C$ *for any* $\lambda\in \mathbb{R}$ *and for* $\eta_{i}= {}^{(a_{i})}{}X_{i}^{(b_{i})}$ *with any* $a_{i}< b_{i}$, $i=1,2, \dots$. (i) *If* $r<\alpha$, *then assume that* ${\mathbb {E}}\vert X\vert^{\alpha}<\infty$. (ii) *If* $r=\alpha$, *then assume that* ${\mathbb {E}}\vert X\vert^{r} \log(1+\vert X \vert) <\infty$. (iii) *If* $r>\alpha$, *then assume that* ${\mathbb {E}}\vert X\vert^{r} <\infty$. *Then*

2.35 $$ \sum_{n=1}^{\infty}n^{\alpha/p-2} {\mathbb {E}}\Biggl\lbrace \Biggl\vert \frac {1}{n^{1/p}} \sum _{k=1}^{n} X_{k} \Biggr\vert -\varepsilon \Biggr\rbrace _{+}^{r} < \infty $$

*for any* $\varepsilon>0$.

#### Proof

Let $t=n^{1/p}$. As ${\mathbb {E}}X_{k} =0$, we have

2.36 $$ \begin{aligned}[b] B \stackrel{{\mathrm{def}}}{=} {}&\sum _{n=1}^{\infty}n^{\alpha/p-2} {\mathbb {E}}\Biggl\lbrace \frac{1}{n^{1/p}} \Biggl\vert \sum_{k=1}^{n} X_{k} \Biggr\vert -\varepsilon \Biggr\rbrace _{+}^{r} \\ ={}&\sum_{n=1}^{\infty}n^{\alpha/p-2} {\mathbb {E}}\Biggl\lbrace \Biggl\vert \frac {1}{n^{1/p}} \sum_{k=1}^{n} \bigl( {}^{(-t)}{}X_{k}^{(t)} - {\mathbb {E}}{}^{(-t)}{}X_{k}^{(t)} \bigr) \\ &{} +\sum_{k=1}^{n} \bigl( {}^{(-\infty )}{}X_{k}^{(-t)} - {\mathbb {E}}{}^{(-\infty)}{}X_{k}^{(-t)} \bigr) \\ &{} +\sum_{k=1}^{n} \bigl( {}^{(t)}{}X_{k}^{(\infty)} - {\mathbb {E}}{}^{(t)}{}X_{k}^{(\infty)} \bigr) \Biggr\vert -\varepsilon \Biggr\rbrace _{+}^{r} \\ \le {}&c \sum_{n=1}^{\infty}n^{\alpha/p-2} {\mathbb {E}}\Biggl( \frac {1}{n^{1/p}} \sum_{k=1}^{n} \bigl( {}^{(-t)}{}X_{k}^{(t)} - {\mathbb {E}}{}^{(-t)}{}X_{k}^{(t)} \bigr) \Biggr)^{2} \\ &{}+ c \sum_{n=1}^{\infty}n^{\alpha/p-2} {\mathbb {E}}\Biggl\vert \frac {1}{n^{1/p}} \sum_{k=1}^{n} \bigl( {}^{(-\infty)}{}X_{k}^{(-t)} - {\mathbb {E}}{}^{(-\infty)}{}X_{k}^{(-t)} \bigr) \Biggr\vert ^{r} \\ &{}+ c \sum_{n=1}^{\infty}n^{\alpha/p-2} {\mathbb {E}}\Biggl\vert \frac {1}{n^{1/p}} \sum_{k=1}^{n} \bigl( {}^{(t)}{}X_{k}^{(\infty)} - {\mathbb {E}}{}^{(t)}{}X_{k}^{(\infty)} \bigr) \Biggr\vert ^{r} , \end{aligned} $$

where we applied Lemma 3.1 of [3]. Now, as $g(n)= C$, by Theorem 2.2 we obtain

2.37 $$ \begin{aligned}[b] B \le {}& c \sum _{n=1}^{\infty}n^{\alpha/p-2} \frac{1}{n^{2/p}} \sum _{k=1}^{n} {\mathbb {E}}\bigl( {}^{(-t)}{}X_{k}^{(t)} - {\mathbb {E}}{}^{(-t)}{}X_{k}^{(t)} \bigr)^{2} \\ &{} + c \sum_{n=1}^{\infty}n^{\alpha/p-2} \frac{1}{n^{r/p}} \sum_{k=1}^{n} {\mathbb {E}}\bigl\vert {}^{(-\infty)}{}X_{k}^{(-t)} - {\mathbb {E}}{}^{(-\infty )}{}X_{k}^{(-t)} \bigr\vert ^{r} \\ &{} + c \sum_{n=1}^{\infty}n^{\alpha/p-2} \frac{1}{n^{r/p}} \sum_{k=1}^{n} {\mathbb {E}}\bigl\vert {}^{(t)}{}X_{k}^{(\infty)} - {\mathbb {E}}{}^{(t)}{}X_{k}^{(\infty)} \bigr\vert ^{r} \\ ={}& T_{2} + T_{1} + T_{3}, \end{aligned} $$

say.

First consider $T_{2}$. Applying Lemma 2.1 and using that $t=n^{1/p}$, we obtain

$$ \begin{aligned} T_{2} & \le c \sum _{n=1}^{\infty}n^{\alpha/p-2/p-2} \sum _{k=1}^{n} {\mathbb {E}}\bigl( {}^{(-t)}{}X_{k}^{(t)} \bigr)^{2} \\ &\le c \sum_{n=1}^{\infty}n^{\alpha/p-2/p-1} {\mathbb {E}}\bigl( {}^{(-t)}{}X^{(t)} \bigr)^{2} \\ &= c \sum_{n=1}^{\infty}n^{\alpha/p-2/p-1} {\mathbb {E}}\vert X \vert ^{2} \mathbb{I}\bigl\{ \vert X \vert \le n^{1/p} \bigr\} + c \sum_{n=1}^{\infty}n^{\alpha/p-2/p-1} \bigl(n^{1/p} \bigr)^{2} \mathbb{P}\bigl\{ \vert X \vert > n^{1/p} \bigr\} \\ & = T_{21} + T_{22}. \end{aligned} $$

Now we have

$$ \begin{aligned} T_{22} & \le c\sum _{n=1}^{\infty}n^{\alpha/p-1} \sum _{k=n}^{\infty} \mathbb{P}\bigl\{ k^{1/p} < \vert X \vert \le(k+1)^{1/p} \bigr\} \\ & = c\sum_{k=1}^{\infty} \mathbb{P}\bigl\{ k^{1/p} < \vert X \vert \le(k+1)^{1/p} \bigr\} \sum _{n=1}^{k} n^{\alpha/p-1} \le c {\mathbb {E}}\vert X \vert ^{\alpha}. \end{aligned} $$

Furthermore,

$$ \begin{aligned} T_{21} & \le c \sum _{n=1}^{\infty}n^{\alpha/p-2/p-1} \sum _{k=1}^{n} {\mathbb {E}}\vert X \vert ^{2} \mathbb{I}\bigl\{ (k-1)^{1/p} < \vert X \vert \le k^{1/p} \bigr\} \\ & = c \sum_{k=1}^{\infty} {\mathbb {E}}\vert X \vert ^{2} \mathbb{I}\bigl\{ (k-1)^{1/p} < \vert X \vert \le k^{1/p} \bigr\} \sum_{n=k}^{\infty}n^{\alpha/p-2/p-1} \le c {\mathbb {E}}\vert X \vert ^{\alpha}. \end{aligned} $$

Therefore we see that $T_{2} <\infty$.

Now, we turn to $T_{1}$ and $T_{3}$. Like in the proof of Theorem 2.6, as $t=n^{1/p}$, we obtain

$$ T_{3} \le c \sum_{n=1}^{\infty}n^{\alpha/p-r/p -2} \sum_{k=1}^{n} {\mathbb {E}}X_{k}^{r} \mathbb{I}\bigl\{ X_{k} > n^{1/p} \bigr\} . $$

Similarly,

$$ T_{1} \le c \sum_{n=1}^{\infty}n^{\alpha/p-r/p -2} \sum_{k=1}^{n} {\mathbb {E}}\vert X_{k} \vert ^{r} \mathbb{I}\bigl\{ X_{k} < -n^{1/p} \bigr\} . $$

Therefore, by Lemma 2.1,

$$ \begin{aligned} T_{1} + T_{3} & \le c \sum _{n=1}^{\infty}n^{\alpha/p-r/p -2} \sum _{k=1}^{n} {\mathbb {E}}\vert X_{k} \vert ^{r} \mathbb{I}\bigl\{ \vert X_{k} \vert > n^{1/p} \bigr\} \\ & \le c \sum_{n=1}^{\infty}n^{\alpha/p-r/p -1} {\mathbb {E}}\vert X \vert ^{r} \mathbb{I}\bigl\{ \vert X \vert > n^{1/p} \bigr\} \\ & \le c \sum_{n=1}^{\infty}n^{\alpha/p-r/p -1} \sum_{k=n}^{\infty} {\mathbb {E}}\vert X \vert ^{r} \mathbb{I}\bigl\{ k^{1/p} < \vert X \vert \le (k+1)^{1/p} \bigr\} \\ & \le c \sum_{k=1}^{\infty} {\mathbb {E}}\vert X \vert ^{r} \mathbb{I}\bigl\{ k^{1/p} < \vert X \vert \le (k+1)^{1/p} \bigr\} \sum_{n=1}^{k} n^{\alpha/p-r/p -1} . \end{aligned} $$

Now, we see the following:

(i) If $r<\alpha$, then $T_{1} + T_{3} \le c {\mathbb {E}}\vert X\vert^{\alpha}<\infty$.
(ii) If $r=\alpha$, then $T_{1} + T_{3} \le c {\mathbb {E}}\vert X\vert^{r} \log (1+\vert X \vert) <\infty$.
(iii) If $r>\alpha$, then $T_{1} + T_{3} \le c {\mathbb {E}}\vert X\vert^{r} <\infty$.

Therefore we see that $B< \infty$ in all cases. □

#### Remark 2.2

For pairwise independent and identically distributed random variables, Theorem 3.7 in [3] states the same assertion as our Theorem 2.7. By our proof we can see that Theorem 3.7 in [3] can be extended to weakly mean dominated pairwise independent random variables. We also see that our Theorem 2.7 implies complete convergence for WOD random variables if $g(n)=C$ in (2.18) and (2.19).

#### Remark 2.3

Under the conditions of Theorem 2.7, we have

2.38 $$ \sum_{n=1}^{\infty}n^{\alpha/p-2} \mathbb{P}\Biggl\lbrace \Biggl\vert \frac {1}{n^{1/p}} \sum _{k=1}^{n} X_{k} \Biggr\vert > \varepsilon \Biggr\rbrace < \infty $$

for any $\varepsilon>0$, which can be proved by usual calculations; see, e.g., [9], Remark 2.6.

## Conclusions

We have obtained general versions of the von Bahr-Esseen moment inequality, the exponential inequality, and convergence theorems. Our results can be applied to prove new limit theorems for weakly dependent sequences.

## References

[1] von Bahr B, Esseen CG (1965). Inequalities for the *r*th absolute moment of a sum of random variables, $1 \leq r \leq2$. Ann. Math. Stat..

[2] Dharmadhikari SW, Jogdeo K (1969). Bounds on moments of certain random variables. Ann. Math. Stat..

[3] Chen P, Bai P, Sung SH (2014). The von Bahr-Esseen moment inequality for pairwise independent random variables and applications. J. Math. Anal. Appl..

[4] Antonini RG, Kozachenko Y, Volodin A (2008). Convergence of series of dependent *φ*-sub-Gaussian random variables. J. Math. Anal. Appl..

[5] Etemadi N (1981). An elementary proof of the strong law of large numbers. Z. Wahrscheinlichkeitstheor. Verw. Geb..

[6] Csörgő S, Tandori K, Totik V (1983). On the strong law of large numbers for pairwise independent random variables. Acta Math. Hung..

[7] Baum LE, Katz M (1965). Convergence rates in the law of large numbers. Trans. Am. Math. Soc..

[8] Chow YS (1988). On the rate of moment convergence of sample sums and extremes. Bull. Inst. Math. Acad. Sin..

[9] Sung SH (2009). Moment inequalities and complete moment convergence. J. Inequal. Appl..

[10] Fuk DH, Nagaev SV (1971). Probabilistic inequalities for sums of independent random variables. Teor. Veroâtn. Primen..

[11] Gan S, Chen P, Qiu D (2011). Rosenthal inequality for NOD sequences and its applications. Wuhan Univ. J. Nat. Sci..

[12] Fazekas, I, Pecsora, S, Porvázsnyik, B: General theorems on exponential and Rosenthal’s inequalities and on complete convergence. Manuscript (2016)

[13] Wang K, Wang Y, Gao Q (2013). Uniform asymptotics for the finite-time ruin probability of a dependent risk model with a constant interest rate. Methodol. Comput. Appl. Probab..

[14] Wang X, Xu C, Hu T-C, Volodin A, Hu S (2014). On complete convergence for widely orthant-dependent random variables and its applications in nonparametric regression models. Test.

[15] Bauer H (1996). Probability Theory.

[16] Gut A (1992). Complete convergence for arrays. Period. Math. Hung..

[17] Fazekas I (1992). Convergence rates in the law of large numbers for arrays. Publ. Math. (Debr.).

