# Erratum to: General Bahr-Esseen inequalities and their applications

**DOI:** 10.1186/s13660-017-1514-9

**Published:** 2017-09-20

**Authors:** István Fazekas, Sándor Pecsora

**Affiliations:** Faculty of Informatics, University of Debrecen, P.O. Box 400, Debrecen, 4002 Hungary

## Erratum

In the publication of this article [1], there were two errors.

1. The error in Section 2.3 Exponential inequalities and their consequences:
  ‘${}^{(a)}{}X^{(b)} = -a \mathbb{I}\{X< a \} + X \mathbb{I}\bigl\{a\le \vert X \vert \le b \bigr\} + b \mathbb{I}\{X> b \}$.’ Should instead read:
  ‘${}^{(a)}{}X^{(b)} = a \mathbb{I}\{X< a \} + X \mathbb{I}\bigl\{a\le X \le b \bigr\} + b \mathbb{I}\{X> b \}$.’
2. The error in Section 2.4 Convergence theorems: ‘A well-known WLLN for pairwise independent r.v.s is the result of Csörgő, Tandori, and Totik [6].’ Should instead read: ‘A well-known SLLN for pairwise independent r.v.s is the result of Csörgő, Tandori, and Totik [6].’

This has now been updated in the original article [1].
